# Racial Differences in Four Leukemia Subtypes: Comprehensive Descriptive Epidemiology

**DOI:** 10.1038/s41598-017-19081-4

**Published:** 2018-01-11

**Authors:** Yinjun Zhao, Yu Wang, Shuangge Ma

**Affiliations:** 1 Yale School of Public Health, 60 College ST, New Haven, CT 06520 USA; 2 School of Statistics and The center for Applied Statistics, Renmin University of China, 59 Zhongguancun Ave., Beijing, 100872 China

## Abstract

Leukemia is a malignant progressive disease and has four major subtypes. Different racial groups differ significantly in multiple aspects. Our goal is to systematically and comprehensively quantify racial differences in leukemia. The SEER database is analyzed, and comprehensive descriptive analysis is provided for the four major subtypes, namely ALL (acute lymphoblastic leukemia), CLL (chronic lymphoblastic leukemia), AML (acute myeloid leukemia), and CML (chronic myeloid leukemia), and for two age groups (≤14 and >14) separately. The racial groups studied include NHW (non-Hispanic White), HW (Hispanic White), BL (Black), and API (Asian and Pacific Islander). Univariate and multivariate analyses are conducted to quantify racial differences in patients’ characteristics, incidence, and survival. For patients’ characteristics, significant racial differences are observed in gender, age at diagnosis, diagnosis era, using radiation for treatment, registry, cancer history, and histology type. For incidence, significant racial differences are observed, and the patterns vary across subtypes, gender, and age groups. For most of the subtypes and gender and age groups, Blacks have the worst five-year survival, and significant racial differences exist. This study provides a comprehensive epidemiologic description of racial differences for the four major leukemia subtypes in the U.S. population.

## Introduction

Leukemia is a cancer of the early blood-forming cells^1^. It is usually a cancer of the white blood cells, although some leukemias start in other blood cell types as well. It is the most common cancer in children and teens, accounting for almost 1 out of 3 cases. In 2016 in the U.S., 60,140 new cases of leukemia and 24,400 deaths are expected. Clinically and pathologically, leukemia can be divided into multiple subtypes^2^. The first division separates leukemias into acute and chronic forms. In acute leukemias, the abnormal blood cells are blast, usually remain immature, and thus fail to carry out their expected functionalities. Acute leukemias are featured with fast development and usually demand immediate attention. In chronic leukemias, there are still some but not exclusive blast cells. However, different from in acute leukemias, these blast cells are more mature and may still function in a normal way. The progression of chronic leukemias is usually much slower and may not need immediate treatment. The second division is based on which kind of blood cell is affected, leading to lymphoblastic and myeloid leukemias. With lymphoblastic leukemia, the malignant changes happen to a type of marrow cells that usually become lymphocytes later on. With myeloid leukemia, the malignant changes happen to a type of marrow cells that usually become red blood cells, some other types of white cells, and platelets later on. The above two divisions lead to a total of four major subtypes: ALL (acute lymphoblastic leukemia), CLL (chronic lymphoblastic leukemia), AML (acute myeloid leukemia), and CML (chronic myeloid leukemia). Beyond these four, there are also other smaller subtypes. The etiology, characteristics, treatment, and survival of childhood and adult leukemias are significantly different^3^, and thus age-specific analysis is usually conducted.

Leukemia occurs in all racial groups. Studies have suggested that different racial groups behave differently in multiple aspects of leukemia. In a study on patients’ characteristics^4^, it is observed that Blacks with CLL had lower median hemoglobin levels, higher beta2-microglobulin levels, and more commonly unmutated IGHV gene, ZAP70 expression, and chromosome 17p or 11q deletion, all of which may contribute to worse outcomes. Another study suggests that racial and ethnic disparities in the incidence and treatment outcomes of childhood ALL persist, with Hispanic children having an elevated risk of developing the disease and one of the lowest survival rates after therapy^5^. The analysis of California Cancer Registry data suggests that Blacks had a lower probability of having chemotherapy, and Blacks and Hispanics had a lower probability of having transplant^6^. For leukemia as well as other cancer types, it has been suggested that differences in treatment exist and may explain some of the observed racial disparities in survival. In a hospital-based study on CLL^4^, it is found that compared to non-Blacks, Blacks had significantly shorter event-free and overall survival. Another study tested the hypothesis that after adjusting for biological factors, Black and Spanish children with newly diagnosed ALL had a worse survival compared to Whites^7^.

A better understanding of racial differences can assist diagnosis, implementation of tailored treatment strategies, and elimination of racial disparity^8,9^. Although sharing the same scheme of analyzing racial differences in leukemia as the aforementioned the other publications, this study may distinguish from them in multiple aspects. First, it analyzes the four major subtypes, four racial groups, and two age groups using the same techniques, facilitates direct comparisons, and is more comprehensive than those that focus on a single subtype/age group and a smaller number of racial groups. Second, the SEER (Surveillance, Epidemiology, and End Results) database is analyzed. The wide coverage and large sample size ensure generalizability and validity and make this study more powerful than those based on a single hospital or community. Third, this study comprehensively addresses patients’ characteristics, incidence, and survival, and can be more comprehensive than those that focus on one single aspect. As such, this study can be complementary to the literature and is warranted.

## Methods

### Study population

Data are obtained from SEER^10^, which is the largest population-based cancer registry in the U.S. and contains input from eighteen regional and state registries. SEER has multiple registry groupings for analysis^11^, which cover different numbers of regions and different time periods. In this study, data are obtained from SEER 13 and 18, which cover approximately 14% and 28% of the U.S. population, respectively. For each case, the first matching record is identified for analysis. In SEER, the four major subtypes are identified by the International Classification of Diseases for Oncology (ICD-O-3) codes and updated by Hematopoietic codes based on the WHO Classification of Tumors of Hematopoietic and Lymphoid, namely

ALL (9826/9835-9836/9811-9818/9837), CLL (9823), AML (9840, 9861, 9865–9867, 9869, 9871–9874, 9895–9897, 9898, 9910–9911, 9920), and CML (9863, 9875–9876, 9945–9946). It is noted that some published studies suggest slightly different definitions. Specifically, they exclude CMML (Chronic Myelomonocytic Leukemia, ICD-O-3 code 9945) from the analysis of CML. We adopt the SEER definition to be coherent with the database. The four major racial groups are HW (Hispanic White), NHW (non-Hispanic White), BL (Black), and API (Asian and Pacific Islander). For the analysis of patients’ characteristics and incidence, SEER 13 contains data on patients diagnosed in the period of 1992–2011 and from thirteen registries. For the analysis of survival, SEER 18 contains data on patients diagnosed in the period of 1992–2006 and followed up to 12/31/2011 and from eighteen registries. Using different registry groupings maximizes sample sizes for analysis.

### Statistical analysis

Data on the four major subtypes and two age groups (≤14 and >14) are analyzed separately. This age division has been suggested in the literature^12,13^. Univariate analyses are conducted to compare patients’ characteristics across racial groups using Chi-squared tests and ANOVA for categorical and continuous variables, respectively. Variables analyzed include gender, age at diagnosis (the younger age group: ≤4, 5–9, 10–14; the older age group: 15–34, 35–54, 55+), diagnosis era (1992–2001, 2002–2011), treatment (no radiation, radiation, unknown), registry (West, Northeast, Midwest, South, which has been suggested in the literature), survival, and histology type. Age-adjusted incidence rates and five-year relative survival rates are computed using the SEER*Stat 8.2.1 software and age-adjusted using the U.S. Census 2000 data as reference. Multivariate Cox regression is then conducted to further investigate racial differences in survival, adjusting for the potential confounding effects of age at diagnosis, gender, diagnosis era, treatment, registry, and histologic type. Analysis that cannot be carried out using SEER*Stat is realized using SAS 9.3 (SAS Institute Inc., Cary, NC, U.S.).

## Results

### Patients’ clinicopathologic characteristics

Results for the age ≤14 group are shown in Table 1. With an insufficient sample size, CLL is not analyzed. For the other three subtypes, there is no difference in the distribution of gender across races. Significant differences in age at diagnosis are observed for ALL (p-value < 0.01). Specifically, APIs have the lowest age at diagnosis, and Blacks have the highest. For ALL, significant differences are also observed for diagnosis era (p-value < 0.001), with more diagnoses in the period of 1992–2001. In the analysis of treatment, as the counts for the “unknown” category are small, only “no radiation” and “radiation” are compared. Significant differences are observed for ALL (p-value 0.035) and CML (p-value < 0.01). Specifically, for ALL, APIs have the highest rate of “no radiation” (92%), while Blacks have the lowest (86.1%). For CML, the trend is reversed, with Blacks having the highest rate of “no radiation” (85.7%) and APIs having the lowest (65%). Significant differences are also observed for registry (p-values < 0.01). For all three subtypes, the dominating majority of HW and API patients are from West, while the percentages of NHW and BL patients from West are much lower. For all subtypes and all racial groups, the dominating majority of patients have no history of cancer. Significant differences in survival are observed for ALL and AML across racial groups (p-values < 0.01). NHWs have the longest mean survival, and BLs have the shortest. For example, for ALL, the mean survival for NHWs and BLs are 94.3 and 82.8 months, respectively. In the analysis of histology type, significant racial differences are observed for ALL and CML (p-value < 0.001 and 0.015, respectively). For ALL, APIs have a lower percentage of “precursor cell LL, NOS” than the other racial groups. For CML, NHWs and APIs have lower percentages of “chronic MML, NOS”.

**Table 1:** Patients’ characteristics and clinicopathologic features (age ≤ 14).

	**ALL**				**AML**				**CML**			
	**HW**	**NHW**	**BL**	**API**	**HW**	**NHW**	**BL**	**API**	**HW**	**NHW**	**BL**	**API**
**Sample**	2066	2851	382	560	295	454	147	122	34	81	14	20
**Gender**												
Male	56	55.2	57.6	57.9	54.6	53.1	51.7	56.6	73.5	66.7	50	75
Female	44	44.8	42.4	42.1	45.4	46.9	48.3	43.4	26.5	33.3	50	25
p-value	0.52				0.77				0.43			
**Age at diagnosis**												
≤4	53.2	56.6	44.5	57.7	46.4	46.9	49.7	49.2	50	43.2	21.4	50
5–9	27.7	27.2	31.7	29.3	21.4	20.7	19.7	19.7	5.9	18.5	21.4	20
10–14	19.1	16.2	23.8	13	32.2	32.4	30.6	31.1	44.1	38.3	57.1	30
p-value	<0.01				0.87				0.43			
**Diagnosis era**												
1992–2001	51.2	58	54.7	51.8	51.5	59	46.3	54.9	44.1	49.4	64.3	55
2002–2011	48.8	42	45.3	48.2	48.5	41	53.7	45.1	55.9	50.6	35.7	45
p-value	<0.001				0.39				0.54			
**Treatment**												
No radiation	88.8	89.6	86.1	92	90.5	88.8	86.4	91	82.4	76.5	85.7	65
Radiation	11	10	13.6	8	9.5	10.8	12.9	8.2	17.6	22.2	14.3	35
Unknown^&^	0.1	0.4	0.3	0	0	0.4	0.7	0.8		1.2		
p-value	0.035				0.94				<0.01			
**Registry**												
West	91.2	56	41.6	90	88.8	57.7	40.8	91	85.3	50.6	50	85
Northeast	3.7	12.2	5.5	1.4	6.1	11	10.2	0.8	8.8	17.3		
Midwest	2.3	24.8	25.7	4.8	2	25.6	18.4	4.1	5.9	24.7	21.4	10
South	2.8	7	27.2	3.8	3.1	5.7	30.6	4.1		7.4	28.6	5
p-value	<0.01				<0.01				<0.01			
**Cancer history**												
No	98.6	98.4	99.5	98.8	96.6	95.6	94.6	91	97.1	95.1	92.9	100
Yes	1.4	1.6	0.5	1.3	3.4	4.4	5.4	9	2.9	4.9	7.1	
p-value	0.38				0.4				0.08			
**Survival (months)**	**79.5 ± 60.7**	**94.3 ± 62.8**	**82.8 ± 63.3**	**84.2 ± 61.4**	**51.7 ± 55**	**63.8 ± 62.1**	**49.1 ± 56.4**	**52.6 ± 56.1**	**47.7 ± 47**	**59 ± 55.4**	**80.9 ± 73**	**71.3 ± 71.3**
p-value	<0.01				<0.01				0.25			
**Histology type**												
Precursor cell LL,NOS	62.5	66.6	63.4	58.8								
Precursor B-cell LL	30.8	27.5	27.2	33.2								
Precursor T-cell LL	3.3	3	6.3	3								
Acute ML NOS					43.7	52.9	56.5	45.9				
Acute PL					11.5	8.8	5.4	10.7				
Acute MML					11.9	8.8	6.8	9				
Chronic ML NOS									64.7	53.1	71.4	55
Chronic MGL									14.7	11.1	21.4	15
Chronic MML,NOS									5.9	6.2		
Others^&^	3.4	2.9	3.1	5	32.9	29.5	31.3	34.4	14.7	29.6	7.1	30
p-value	<.001				0.09				0.015			

Cancers diagnosed in the period of 1992–2011 in the SEER 13 database. For a continuous variable, mean ± SD; For a categorical variable, percentage.

^&^This category contains other minor histologic types, which are not included when calculating p-values.

^#^Only five cases of CLL.

Results for the age > 14 group are shown in Table 2. For AML, the distribution of gender differs significantly across races (p-value < 0.01). NHWs have the highest percentage of male (54.7%), while BLs having the lowest (49.1%). For all subtypes, the distribution of age at diagnosis differs significantly across races (p-values < 0.01). NHWs have the highest age at diagnosis, whereas HWs, BLs, HWs, and HWs have the lowest for the four subtypes, respectively. Diagnosis era has significant racial differences for all four subtypes. Specifically, the racial groups that have more diagnoses in the 1992–2001 period are NHW (ALL), NHW and BL (CLL), NHW and BL (AML), and NHW (CML), respectively. For treatment, significant racial differences are observed for AML and CML, although it is noted that the dominating majority had “no radiation”. Significant racial differences are observed for registry, and the patterns are similar to those in Table 1. Unlike for the younger age group, significantly more patients have a history of cancer, and racial differences are significant for all subtypes, with NHWs having the highest percentages of cancer history. Significant racial differences are observed for survival time, and patterns vary across subtypes. For example, for ALL, APIs have the longest mean survival (36.7 months), while BLs have the lowest (24.3 months). In comparison, for CLL, NHWs have the longest mean survival (59.3 months), while BLs have the lowest (50.6 months). For ALL, AML, and CML, racial differences are observed for histology type (p-values < 0.01). For example, for ALL, the percentage of NHWs with “precursor cell LL, NOS” (64.9%) is much higher than that for HWs (53.5%).

**Table 2:** Patients’ characteristics and clinicopathologic features (age > 14).

	**ALL**				**CLL**				**AML**				**CML**			
	**HW**	**NHW**	**BL**	**API**	**HW**	**NHW**	**BL**	**API**	**HW**	**NHW**	**BL**	**API**	**HW**	**NHW**	**BL**	**API**
**Sample**	1438	2705	332	466	1285	24620	1835	718	2393	16565	1747	2224	1156	7874	974	864
**Gender**																
Male	58.9	57.5	53	55.6	57.1	59.5	57.8	62.3	53.4	54.7	49.1	51.7	58.7	58	56.9	61.7
Female	41.1	42.5	47	44.4	42.9	40.5	42.2	37.7	46.6	45.3	50.9	48.3	41.3	42	43.1	38.3
p-value	0.24				0.06				<0.01				0.28			
**Age at diagnosis**																
15–34	55.2	30.8	38.3	35.4	0.5	0.2	0.4	0.4	24.1	6.2	11	12.4	20.8	6	13.1	14.6
35–54	24.9	24.7	30.4	28.5	13.1	10.4	14.9	14.5	26.7	15.8	25.4	23.4	34.4	19.4	31.4	29.7
≥55	19.9	44.5	31.3	36.1	86.4	89.3	84.6	85.1	49.1	78	63.6	64.2	44.8	74.7	55.4	55.7
p-value	<0.01				<0.01				<0.01				<0.01			
**Diagnosis era**																
1992–2001	43.3	54.1	46.7	46.4	45.4	50.4	50.9	41.2	47.3	54.5	50.1	47.7	51.4	55.3	52.5	51.3
2002–2011	56.7	45.9	53.3	53.6	54.6	49.6	49.1	58.8	52.7	45.5	49.9	52.3	48.6	44.7	47.5	48.7
p-value	<0.01				<0.01				<0.01				0.012			
**Treatment**																
No radiation	75.8	77.4	80.1	75.1	96.9	97.2	97.5	94.6	92.1	94.2	95.1	92.8	92.8	94.8	96	92.6
Radiation	22.7	20.8	17.8	22.1	0.9	0.7	0.8	0.6	6.1	4	3.3	5.6	5.2	3.1	2.5	4.4
Unknown**	1.5	1.7	2.1	2.8	2.3	2.2	1.6	4.9	1.7	1.9	1.6	1.6	2	2.1	1.5	3
p-value	0.3				0.7				<0.01				<0.01			
**Registry**																
West	91.8	55	45.5	89.9	90	57.1	44	95.1	88.3	54.9	44.6	94.1	90	56	41.5	92.9
Northeast	4	12.7	6.9	3.6	4.6	11.3	6.3	0.8	5.8	12	7.4	1.3	4.9	10.7	6.2	1.5
Midwest	1.6	25.5	21.1	3	3.4	27.4	32.6	3.3	3.3	27.2	29.1	2.3	2.8	28.6	30.8	3.6
South	2.6	6.8	26.5	3.4	2	4.3	17.2	0.7	2.7	5.9	18.9	2.3	2.3	4.7	21.6	2
p-value	<0.01				<0.01				<0.01				<0.01			
**Cancer history**																
No	92.7	84.6	87.7	89.9	75.8	69.3	68.9	74.9	85.5	75	76.9	83.3	88.4	76.7	80.4	83.4
Yes	7.3	15.4	12.3	10.1	24.2	30.7	31.1	25.1	14.5	25	23.1	16.7	11.6	23.3	19.6	16.6
p-value	<0.01	<0.01	<0.01	<0.01												
**Survival (months)**	34 ± 43.8	35.6 ± 50.5	24.3 ± 35.1	36.7 ± 49.5	53.1 ± 48.3	59.3 ± 50.4	50.6 ± 44.7	55.6 ± 51.2	25.6 ± 42.7	17.5 ± 36.2	18.7 ± 37	22.3 ± 40.9	49.2 ± 49.9	40.6 ± 46.3	42.8 ± 45.9	47.9 ± 49.9
p-value	<0.01				<0.01				<0.01				<0.01			
**Histology type**																
Precursor cell LL,NOS	53.5	64.9	61.7	58.8												
Precursor B-cell LL	39.4	24.9	23.8	28.1												
Precursor T-cell LL	3	3.6	7.8	7.7												
Acute ML NOS									51.5	60.7	61.5	56.6				
Acute PL									14.7	6	8.3	7.5				
Acute MML									10.4	10.4	9.9	9.5				
Chronic ML NOS													72.2	63.7	73.7	67.1
Chronic MGL													13.2	7.7	10.8	11.6
Chronic MML,NOS													14.4	28.3	15.3	20.5
Others^&^	4.2	6.6	6.6	5.4					23.5	22.8	20.3	26.4	0.2	0.3	0.2	0.8
p-value	<0.01								<0.01				<0.01			

Cancers diagnosed in the period of 1992–2011 in the SEER 13 database. For a continuous variable, mean ± SD; For a categorical variable, percentage.

LL: lymphoblastic leukemia; ML: myeloid leukemia; PL: promyelocytic leukemia; MML: myelomonocytic leukemia; MGL: chronic myelogenous leukemia, BCR/ABL positive.

^&&^This category contains other minor histologic types, which are not included when calculating p-values.

**Figure 1:**
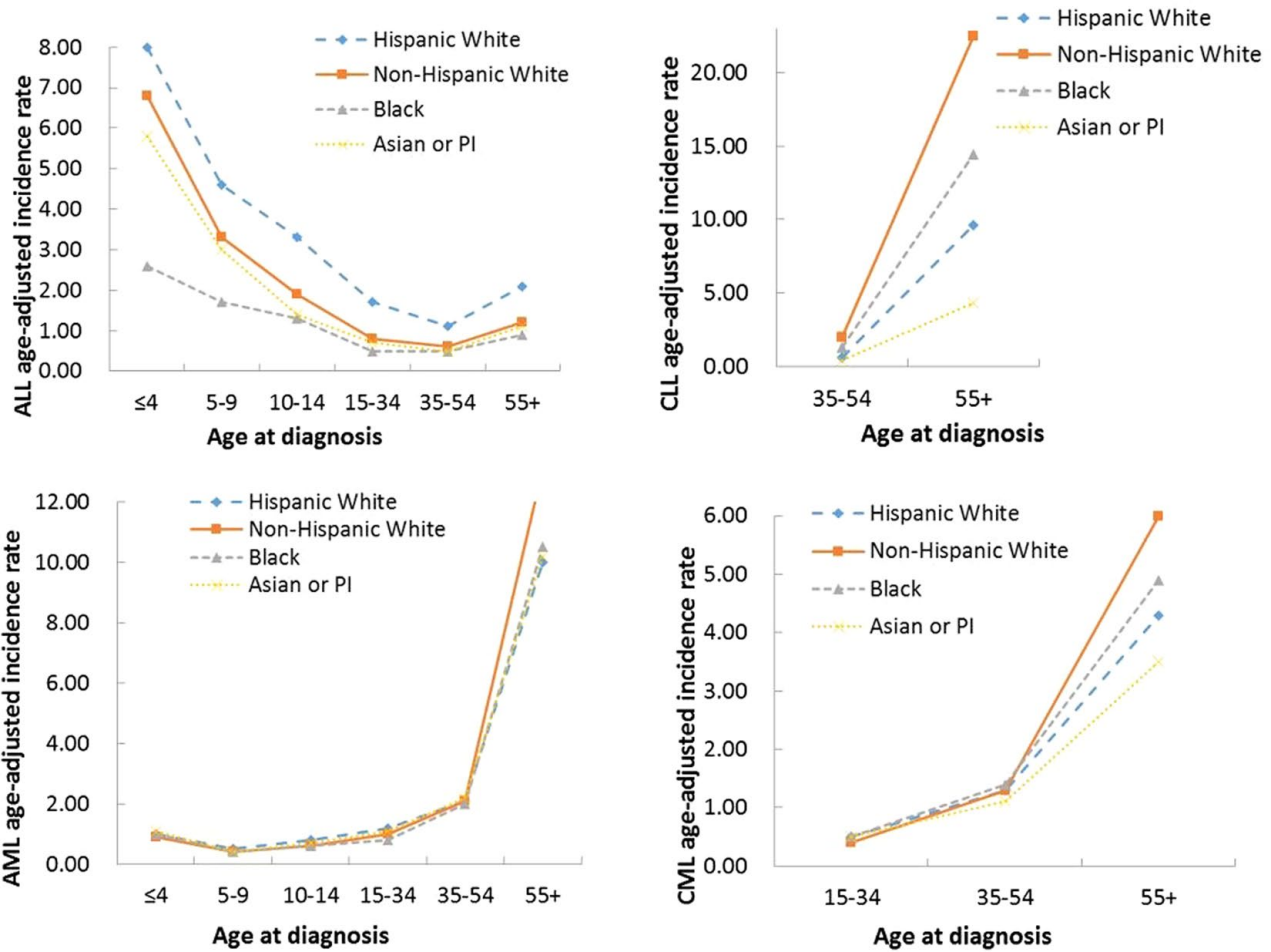
Age-adjusted incidence rates per 100,000 person-years. Notes: Only five cases with CLL and age ≤ 14. Cancers diagnosed in the period of 1992–2011 in the SEER 13 database. Rates are age-standardized using the U.S. Census 2000 population as reference.

The age-adjusted incidence rates are presented in Table 3. Some analyses are not conducted because of small counts. For the ≤14 age group, for ALL, HWs have the highest incidence rate (5.3 per 100,000 person-years), followed by NHWs (3.9) and APIs (3.3), while BLs have the lowest incidence (1.9). When stratified by gender and age, the patterns persist, although the relative magnitudes are different. For AML, the overall incidence rate is much lower, and different racial groups have similar low rates. When stratified by gender and age, the patterns are similar. In the analysis of CML, the overall rate is low, and most counts are too small to generate reliable estimates. For the >14 age group, for ALL, HWs have the highest incidence rate (1.6 per 100,000 person-years), NHWs and APIs have comparable rates (0.8), and BLs have the lowest (0.6). The patterns are mostly retained in the stratified analysis. For CLL, overall, NHWs have the highest incidence rate (6.9), followed by BLs (4.4) and then HWs (2.9). Males have much higher incidence rates, and the racial patterns for both males and females are similar to those overall. A significant dependence on age is observed, with the 55+ age group having a much higher incidence. In the analysis of AML, overall, NHWs have a higher incidence rate, while the other three races have similar rates. Males have higher incidence rates, and the across-race patterns for both males and females are similar to those overall. The dependence on age is observed, with the 55+ group having a significantly higher incidence. For CML, overall, NHWs have the highest rate (2.2), followed by BLs (2.0) and then NHs (1.8). Similar patterns are observed for both males and females, with males having a higher incidence. An increasing trend with age is observed. For a better visualization, the incidence analysis results are also shown in Fig. 1.

**Table 3:** Age-adjusted incidence rates per 100,000 person-years.

	**Age ≤ 14**				**Age > 14**				
	**HW**	**NHW**	**BL**	**API**		**HW**	**NHW**	**BL**	**API**
**ALL**									
Total	5.3(5,5.5)	3.9(3.8,4.1)	1.9(1.7,4.1)	3.3(3.1,3.6)	Total	1.6(1.5,1.7)	0.8(0.8,0.9)	0.6(0.6,0.9)	0.8(0.7,0.8)
Male	5.8(5.4,6.1)	4.2(4,4.4)	2.2(1.9,4.4)	3.7(3.3,4.1)	Male	1.8(1.6,1.9)	1(1,1.1)	0.7(0.6,1.1)	0.9(0.8,1)
Female	4.7(4.5,5)	3.6(3.4,3.8)	1.6(1.4,3.8)	2.9(2.6,3.3)	Female	1.4(1.3,1.5)	0.7(0.6,0.7)	0.6(0.5,0.7)	0.6(0.6,0.7)
≤4	8(7.5,8.4)	6.8(6.5,7.1)	2.6(2.3,7.1)	5.8(5.2,6.4)	15–34	1.7(1.6,1.8)	0.8(0.8,0.9)	0.5(0.4,0.9)	0.7(0.6,0.8)
5–9	4.6(4.3,5)	3.3(3.1,3.5)	1.7(1.5,3.5)	3(2.5,3.4)	35–54	1.1(1,1.3)	0.6(0.5,0.6)	0.5(0.4,0.6)	0.5(0.5,0.6)
10–14	3.3(3,3.6)	1.9(1.7,2.1)	1.3(1.1,2.1)	1.4(1.1,1.7)	55+	2.1(1.8,2.3)	1.2(1.2,1.3)	0.9(0.8,1.3)	1.1(1,1.3)
**CLL** ^*^									
Total	—	—	—	—	Total	2.9(2.7,3)	6.9(6.8,7)	4.4(4.2,7)	1.4(1.3,1.4)
Male	—	—	—	—	Male	3.9(3.6,4.2)	9.6(9.4,9.7)	6.3(5.9,9.7)	1.9(1.8,2.1)
Female	—	—	—	—	Female	2.1(2,2.3)	4.9(4.8,5)	3.2(3,5)	0.9(0.8,1)
≤4	—	—	—	—	15–34	—	—	—	—
5–9	—	—	—	—	35–54	0.6(0.6,0.7)	2(2,2.1)	1.3(1.2,2.1)	0.4(0.4,0.5)
10–14	—	—	—	—	55+	9.6(9,10.1)	22.5(22.2,22.8)	14.4(13.7,22.8)	4.3(4,4.7)
**AML**									
Total	0.8(0.7,0.8)	0.6(0.6,0.7)	0.7(0.6,0.7)	0.7(0.6,0.9)	Total	3.9(3.8,4.1)	4.7(4.6,4.8)	3.9(3.7,4.8)	4(3.8,4.1)
Male	0.8(0.7,1)	0.6(0.6,0.7)	0.7(0.5,0.7)	0.8(0.6,1)	Male	4.8(4.5,5.1)	5.9(5.8,6)	4.7(4.4,6)	4.7(4.5,5)
Female	0.7(0.6,0.8)	0.6(0.5,0.7)	0.7(0.5,0.7)	0.7(0.5,0.9)	Female	3.4(3.2,3.6)	3.9(3.8,3.9)	3.4(3.2,3.9)	3.4(3.2,3.6)
≤4	1(0.8,1.2)	0.9(0.8,1)	1(0.8,1)	1.1(0.8,1.4)	15–34	1.2(1.1,1.3)	1(0.9,1)	0.8(0.7,1)	1.1(1,1.2)
5–9	0.5(0.4,0.6)	0.4(0.3,0.5)	0.4(0.3,0.5)	0.4(0.3,0.6)	35–54	2.1(2,2.3)	2.1(2,2.2)	2(1.9,2.2)	2.2(2,2.4)
10–14	0.8(0.6,1)	0.6(0.5,0.7)	0.6(0.4,0.7)	0.7(0.5,0.9)	55+	10(9.5,10.6)	13.1(12.9,13.3)	10.5(9.9,13.3)	10.2(9.7,10.7)
**CML**									
Total	0.1(0.1,0.1)	0.1(0.1,0.1)	—	—	Total	1.8(1.7,2)	2.2(2.2,2.3)	2(1.9,2.3)	1.5(1.4,1.6)
Male	0.1(0.1,0.2)	0.1(0.1,0.2)	—	—	Male	2.4(2.2,2.6)	3(2.9,3)	2.7(2.5,3)	2.1(1.9,2.3)
Female	—	0.1(0,0.1)	—	—	Female	1.4(1.3,1.6)	1.7(1.6,1.8)	1.5(1.4,1.8)	1.1(1,1.2)
≤4	—	—	—	—	15–34	0.5(0.5,0.6)	0.4(0.4,0.5)	0.5(0.4,0.5)	0.5(0.4,0.6)
5–9	—	—	—	—	35–54	1.3(1.2,1.4)	1.3(1.2,1.3)	1.4(1.3,1.3)	1.1(0.9,1.2)
10–14	—	—	—	—	55+	4.3(4,4.7)	6(5.8,6.1)	4.9(4.5,6.1)	3.5(3.2,3.8)

Cancers diagnosed in the period of 1992–2011 in the SEER 13 database. In each cell, estimate (95% CI). Rates are age-standardized using the U.S. Census 2000 population as reference. Statistics are not displayed when there are fewer than 25 cases or the population size is less than 50,000.

**Figure 2:**
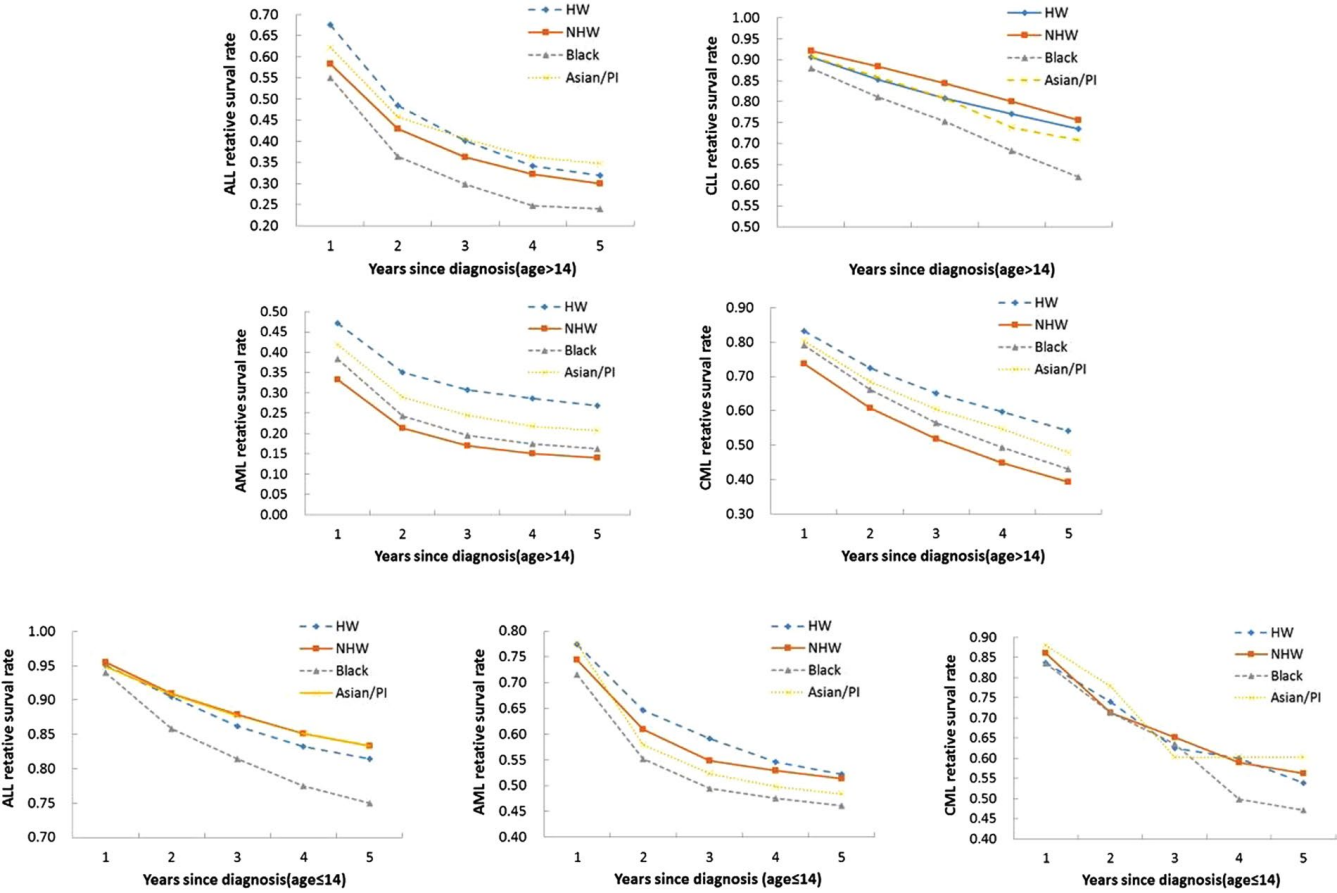
Age-adjusted relative survival rates up to five years. Cancers diagnosed in the period of 1992–2006 and followed up to 12/31/2011 in the SEER 18 database. Rates are age-standardized using the U.S. Census 2000 population as reference. Analysis is not conducted for CLL and age ≤ 14, which has only five cases.

The five-year relative survival rates are provided in Table 4, along with the p-values of race from the multivariate Cox regression analysis. Analysis is not conducted for the CML age ≤14 group because of a small sample size. The detailed Cox regression results are available from the authors. The relative survival rates are also calculated for up to five years, and the results for all races and subtypes are presented in Fig. 2. For the ≤14 age group, in the analysis of ALL, NHWs and APIs have the highest five-year survival rates (0.83), while BLs have the lowest (0.75). In the multivariate analysis, after adjusting for confounding effects, the racial difference is significant (p-value < 0.01). In the stratified analyses, the patterns are mostly consistent, with the exception that for the 5–9 years age group, APIs have the lowest survival rate (0.78). In all of the stratified analyses, the racial differences are statistically significant. For AML overall, HWs have the highest survival rate (0.52), BLs have the lowest (0.46), and the racial difference is significant. Different patterns are observed for males and females. The racial difference is significant in multivariate analysis for females but not males. When stratified by age, only the 10–14 years age group has a significant difference (p-value 0.049). In the analysis of CML, overall, APIs have the highest survival rate (0.6), followed by NHWs (0.56), while BLs have the lowest (0.47). Different patterns are observed for males and females. Specifically, the female HWs have a much higher survival rate, although it is noted that this result should be interpreted cautiously because of the small sample size. When stratified by age, different patterns are observed for different age groups. For the >14 age group, for ALL overall, APIs have the highest survival rate (0.35), while BLs have the lowest, and the racial difference is significant (p-value < 0.01). When stratified by gender, similar patterns are observed, and both gender groups demonstrate significant racial differences. When stratified by age, significant racial differences are observed for the 15–34 and 35–54 age groups, and the patterns are different. Specifically, for these two age groups, NHWs and APIs have the highest survival rates, respectively. For CLL, significant racial differences are observed for overall and most of the stratified analyses, with the exception of age group 15–34. In those analyses with significant racial differences, BLs are observed to have lower survival rates. For example, for overall, BLs have a five-year survival rate of 0.62, compared to 0.74 (HW), 0.76 (NHW), and 0.71 (API). For AML overall, HWs have the highest five-year survival rate (0.27), while NHWs have the lowest (0.14), and the racial difference is significant (p-value < 0.01). For both males and females and for the 35–54 and 55+ age groups, the racial differences are significant. However, the patterns differ across subgroups. For CML, overall, HWs have the highest survival rate (0.54), NHWs have the lowest (0.39), and the racial difference is significant. Significant differences are also observed in the stratified analysis for females, 35–54, and 55+ age groups. For females and the 55+ age group, NHWs have the lowest survival rates, while for the 35–54 age group, BLs have the lowest rate. Figure 2 shows that the patterns are mostly persistent across time. However, a few “crossings” are observed, for example, for the CML ≤ 14 age group.

**Table 4:** Five-year relative survival rates.

	Age≤14					Age>14					
	HW	NHW	BL	API	P-value		HW	NHW	BL	API	P-value
**ALL**											
Total	0.81(0.8,0.83)	0.83(0.82,0.84)	0.75(0.72,0.78)	0.83(0.81,0.86)	<0.01	Total	0.32(0.3,0.34)	0.3(0.29,0.31)	0.24(0.2,0.31)	0.35(0.31,0.39)	<0.01
Male	0.81(0.79,0.83)	0.82(0.8,0.83)	0.74(0.7,0.78)	0.81(0.77,0.84)	<0.01	Male	0.34(0.31,0.37)	0.3(0.29,0.32)	0.24(0.19,0.32)	0.35(0.3,0.4)	<0.01
Female	0.82(0.8,0.84)	0.85(0.84,0.87)	0.76(0.71,0.8)	0.87(0.83,0.9)	0.012	Female	0.29(0.25,0.32)	0.29(0.27,0.31)	0.24(0.19,0.31)	0.34(0.28,0.4)	<0.01
≤4	0.84(0.82,0.86)	0.86(0.84,0.87)	0.75(0.7,0.8)	0.88(0.85,0.91)	<0.01	15-34	0.43(0.39,0.46)	0.5(0.48,0.53)	0.36(0.3,0.53)	0.47(0.4,0.53)	<0.01
5–9	0.83(0.8,0.86)	0.85(0.83,0.87)	0.81(0.75,0.86)	0.78(0.72,0.83)	<0.01	35-54	0.23(0.19,0.27)	0.28(0.25,0.31)	0.19(0.13,0.31)	0.36(0.29,0.43)	0.047
10–14	0.7(0.66,0.74)	0.73(0.7,0.76)	0.67(0.59,0.73)	0.73(0.64,0.8)	0.005	55+	0.11(0.07,0.15)	0.12(0.1,0.14)	0.09(0.04,0.14)	0.15(0.1,0.22)	0.726
**CLL**											
Total						Total	0.74(0.7,0.76)	0.76(0.75,0.76)	0.62(0.6,0.76)	0.71(0.66,0.75)	<0.01
Male						Male	0.69(0.65,0.73)	0.75(0.74,0.75)	0.59(0.56,0.75)	0.7(0.64,0.75)	<0.01
Female						Female	0.79(0.74,0.83)	0.77(0.76,0.78)	0.66(0.62,0.78)	0.71(0.64,0.77)	<0.01
≤4						15–34	0.79(0.47,0.93)	0.76(0.66,0.84)	0.63(0.37,0.84)	0.5(0.01,0.91)	0.359
5–9						35–54	0.87(0.81,0.91)	0.88(0.87,0.89)	0.72(0.67,0.89)	0.89(0.8,0.94)	<0.01
10–14						55+	0.71(0.67,0.74)	0.74(0.73,0.75)	0.6(0.57,0.75)	0.68(0.63,0.72)	<0.01
**AML**											
Total	0.52(0.47,0.57)	0.51(0.48,0.55)	0.46(0.4,0.52)	0.48(0.41,0.55)	<0.01	Total	0.27(0.25,0.29)	0.14(0.14,0.14)	0.16(0.15,0.18)	0.21(0.19,0.23)	<0.01
Male	0.52(0.45,0.59)	0.5(0.45,0.54)	0.48(0.39,0.57)	0.46(0.36,0.56)	0.051	Male	0.26(0.24,0.28)	0.13(0.12,0.13)	0.16(0.14,0.13)	0.18(0.16,0.2)	<0.01
Female	0.52(0.44,0.59)	0.54(0.49,0.58)	0.44(0.35,0.52)	0.51(0.4,0.61)	0.011	Female	0.28(0.25,0.3)	0.16(0.15,0.16)	0.17(0.14,0.16)	0.24(0.21,0.27)	<0.01
≤4	0.55(0.48,0.62)	0.52(0.47,0.56)	0.46(0.36,0.55)	0.48(0.38,0.58)	0.098	15–34	0.48(0.44,0.51)	0.41(0.39,0.44)	0.32(0.28,0.44)	0.41(0.36,0.46)	0.084
5–9	0.49(0.38,0.6)	0.55(0.48,0.61)	0.54(0.41,0.66)	0.53(0.37,0.67)	0.434	35–54	0.36(0.33,0.4)	0.31(0.3,0.33)	0.28(0.24,0.33)	0.35(0.31,0.38)	0.037
10–14	0.5(0.4,0.58)	0.49(0.44,0.54)	0.41(0.31,0.51)	0.45(0.31,0.58)	0.049	55+	0.09(0.07,0.11)	0.07(0.06,0.07)	0.07(0.05,0.07)	0.1(0.08,0.11)	<0.01
**CML**											
Total	0.54(0.39,0.67)	0.56(0.48,0.64)	0.47(0.31,0.62)	0.6(0.41,0.75)		Total	0.54(0.51,0.57)	0.39(0.38,0.4)	0.43(0.4,0.46)	0.48(0.45,0.51)	<0.01
Male	0.43(0.26,0.59)	0.54(0.43,0.64)	0.53(0.29,0.71)	0.58(0.35,0.76)		Male	0.54(0.5,0.57)	0.39(0.38,0.4)	0.41(0.37,0.44)	0.48(0.44,0.53)	0.216
Female	0.72(0.43,0.88)	0.6(0.46,0.71)	0.41(0.19,0.62)	0.66(0.26,0.88)		Female	0.55(0.51,0.59)	0.4(0.39,0.42)	0.46(0.42,0.5)	0.47(0.42,0.52)	<0.01
≤4	0.46(0.29,0.62)	0.57(0.44,0.68)	0.32(0.09,0.58)	0.62(0.33,0.81)		15–34	0.66(0.6,0.71)	0.59(0.56,0.63)	0.56(0.5,0.62)	0.66(0.58,0.73)	0.134
5–9	0.5(0.06,0.85)	0.56(0.36,0.72)	0.48(0.18,0.72)	0.5(0.06,0.85)		35–54	0.64(0.59,0.68)	0.61(0.59,0.63)	0.55(0.5,0.59)	0.63(0.57,0.68)	<0.01
10–14	0.66(0.34,0.85)	0.56(0.42,0.68)	0.57(0.3,0.77)	0.62(0.27,0.84)		55+	0.39(0.34,0.43)	0.3(0.29,0.31)	0.31(0.27,0.34)	0.32(0.28,0.37)	0.035

Cancers diagnosed in the period of 1992–2006 and followed up to 12/31/2011 in the SEER 18 database. In each cell, estimated rate (95% CI). P-values were obtained from multivariate Cox regression. Rates are age-standardized using the U.S. Census 2000 population as reference.

## Discussion

### Findings

As a major cancer type, the epidemiology of leukemia has been extensively studied, and it has been well acknowledged that racial differences exist in multiple aspects of leukemia. However, most of the existing studies simply include race as a confounder in analysis. A few studies have been focused on racial differences, however, usually limited to the disease overall or a single subtype, fewer racial groups, and one single aspect of the disease. This article has filled the knowledge gap by comprehensively examining racial differences for the four major subtypes, multiple aspects of the disease (including patients’ characteristics, incidence, and survival), and four major racial groups. Analyzing data on the same ground using the same techniques allows for direct across-analysis comparisons. It should be noted that this study and some published ones may have analyzed different datasets/time periods, which may lead to results not directly comparable. With the broad coverage of the analyzed SEER data and wide timespan, findings made in this study may complement the existing literature. U.S. is an immigrant country with a significant racial mixture. Observations made in this study can provide more detailed information than the existing literature and assist public health and clinical investigators to better understand this disease, develop and implement tailored treatment and health care programs, and more appropriately allocate medical resources.

Different from most other cancer types, leukemia is a major cancer for children (although the overall rate is still low). Childhood and adult leukemias differ in multiple aspects, and so analysis has been conducted for different age groups separately. Similar to other cancers, racial differences have been observed in multiple patients’ characteristics. For the ≤14 age group, data on ALL, AML, and CML have been analyzed, and racial differences have been observed in the distributions of age at diagnosis, diagnosis era, treatment, registry, survival, and histology type. It is noted that, as there is only a small number of CML cases, the corresponding results should be interpreted with cautions. For ALL, the higher age at diagnosis for BLs may be caused by later onset, later diagnosis, and other factors, as have been suggested in the literature for other cancers. More data collection is needed to “tease out” the effects of, for example, later onset and quantify the extent of later diagnosis, and prevention and control programs should be developed accordingly to eliminate such disparity. The differences in diagnosis era can be confounded by differences in population structure and should be interpreted with cautions. Comparatively, there are fewer NHW ALLs in the 2002–2011 period, which can be caused by changes in both incidence and diagnosis. There are multiple treatment strategies for leukemia^14,15^, including induction chemotherapy, consolidation therapy (or intensification therapy), preventive therapy, maintenance treatments with chemotherapeutic drugs, and others. For most cases, radiation is not the mainline treatment, as has been observed in this analysis. Unfortunately, SEER only provides limited information on treatment. Similar problems have been observed in other racial difference studies using SEER. Some smaller studies have more informatively examined treatment. For example, a California Cancer Registry-based study^6^ found that the Black race was associated with a lower probability of chemotherapy, and Blacks and Hispanics had a lower probability of transplant. The significant racial differences in registry are attributable to the uneven racial distribution of the U.S. population. In the analysis of histology type, the difference observed for CML again needs to be interpreted cautiously because of the small counts. For ALL, APIs have a lower percentage of precursor cell LL, NOS, which is likely caused by genetic factors. In the analysis of the >14 age group, more racial differences are observed. In particular, the distribution of gender is found to differ across races for AML, and the distribution of cancer history differs across races for all four subtypes. The cause of leukemia is still not completely known. The observed difference in gender distribution can be caused by genetic factors (that are related to gender)^16^ as well as gender-related confounders such as smoking, occupational exposure to radiation and chemicals, which contribute to leukemia risk, and others. Unlike for the younger age group, a higher percentage of cancer history is observed. The significantly higher percentage for NHWs can be caused by both genetic factors (that lead to cancer co-occurrence^17^) as well as a higher rate of diagnosis.

The incidence of leukemia is extremely complex. The observed incidence rates depend on the actual incidence as well as diagnosis and reporting. Multiple risk factors for leukemia overall and subtypes have been suggested in the literature, although the exact cause of leukemia is still not fully understood. In addition, it has been suggested that different subtypes, with their different pathological behaviors, have significantly different sets of risk factors^15^. Risk factors that have been suggested for leukemia overall and/or specific subtypes include smoking, exposure to chemicals, history of cancer and treatment, exposure to radiation, certain blood problems, congenital syndromes, family history, viral infections, as well as genetic abnormalities. Many of these factors, for example smoking, exposure to chemicals and radiation, and cancer history and treatment, have been suggested as race-dependent. A few recent small-scale studies have also reported variations of molecular risk factors across races. For example, a recent study was focused on racial differences in CLL and examined genes Notch 1, SF3B1, p53, MyD88, BIRC3, ZAP70, and SCF^18^. Another study examined Black patients with CLL and suggested that they were more likely to be presented with unmutated IGHV gene, ZAP70 expression, and chromosome 17p or 11q deletion^4^. In the literature, although multiple recent studies have investigated genetic, epigenetic, and genomic markers for leukemia etiology^19,20^, attention to their racial differences or subtype- and race-specific interactions between genetic and other risk factors is still insufficient. Another limitation of the existing molecular studies on etiology is their insufficient power (small sample sizes).

The prognosis of leukemia overall and subtypes has been studied extensively in the literature^21,22^. Quite a few studies have suggested a survival disadvantage of the Blacks^4,6,23^. In our analysis, the survival disadvantage of the Blacks is observed for most but not all of the subtypes and age/gender groups. Multiple factors have been suggested to contribute to prognosis. One study suggested that the poor prognosis of Black children with AML was attributable to excessive treatment related mortality but not baseline differences in disease characteristics, response to therapy, or complications from stem cell transplant^23^. Another study suggested that the survival disadvantage of Black women with CML could be caused by selective imatinib resistance, which is likely to be caused by genetic factors^24^. In our analysis, it is observed that AML and CML have the worst prognosis in adult NHWs. This observation is consistent with the literature, where APL (acute promyelocytic leukemia), which has better prognosis than other subtypes, was excluded^25^. The poor prognosis of NHWs and Blacks with AML may be attributable to their higher rates of previous cancers (including previous AML, which may lead to a higher risk of secondary AML). For AML, prognostic factors suggested in the literature include treatment-related factors, for example, Zubrod scale^26,27^, age, serum albumin, and bilirubin, and resistance-to-treatment-related factors, of which the most important ones are the pretreatment cytogenetic and molecular genetic markers in AML blast. For CML, prognosis in the early chronic stage is analytically determined by scores derived from clinical and laboratory features. Other factors that have been suggested as associated with prognosis include cytogenetic changes, for example, deletions of the derivative chromosome 9, and degree and timing of hematological, cytogenetic and molecular responses. The poor prognosis of NHW can be caused by one or multiple of these factors, for example, diagnosis at older ages. In the literature, there is still a lack of study linking, for example, the aforementioned genetic risk factors with the NHW race. A limitation of SEER is that it does not have detailed information on treatment, which can be strongly associated with survival and also vary across races. Another factor that is also associated with survival and may vary across races is socioeconomic status^28^, which may directly affect early diagnosis, ready access to quality health care, and sufficient time and energy to maintain compliance with treatment. SEER started to have insurance information in 2007. For the analyzed time period, linking to other databases for example Medicare, is needed to obtain more useful information on treatment and socioeconomic status. In recent omics studies, molecular changes have also been associated with survival^22^. Similar to etiology, variations of molecular risk factors across races have not been carefully investigated for prognosis.

### Limitations

SEER is chosen as the source of data because of its comprehensiveness, wide coverage, and large sample size. On the other hand, its limitations have been well noted. Specifically, important information, for example on treatment, socioeconomic status, environmental exposures, and genetic risk factors, is missing. Smaller hospital-based studies and linking with other databases may solve some of the problems, but they also have limitations such as small sample sizes and biased sample selection. Another complication may be brought by the multiple coexisting classification schemes. The old SEER database used the ICD-O coding, which was later converted to ICD-O-3, causing unclassified cases. The SEER population also have a higher proportion of foreign-born patients than the general U.S. population. Patients’ characteristics, incidence, and survival all depend on environmental and socioeconomic factors, which vary significantly across countries. The analysis results drawn on the U.S. population may not be generalized to other countries.

### Summary

This study has conducted an epidemiologic analysis and quantified racial differences for four major leukemia subtypes, four racial groups, and two age groups in multiple aspects. It advances from the existing literature by being more comprehensive. Some plausible causes of the observed differences have been suggested. It is also noted that the SEER database is limited by lacking certain important information. More comprehensive data collection and analysis are needed to fully decipher the observed racial differences. Despite certain limitations, as shown in the published SEER-based studies, it is expected that this study can be useful to public health and medical investigators by assisting in early detection, risk stratification, proper treatment selection, and ultimately elimination of racial disparity in leukemia.
